# Intestinal obstruction due to internal hernia with dual defects following laparoscopic retroperitoneal tumor resection: a case report

**DOI:** 10.1097/RC9.0000000000000546

**Published:** 2026-06-02

**Authors:** Kenichiro Yambe, Shingo Tsujinaka, Kuniharu Yamamoto, Kento Morozumi, Chikashi Shibata, Yu Katayose

**Affiliations:** a Division of Hepato-Biliary and Pancreatic Surgery, Department of Surgery, Tohoku Medical and Pharmaceutical University, Sendai, Japan; b Division of Gastroenterologic Surgery, Department of Surgery, Tohoku Medical and Pharmaceutical University, Sendai, Japan; c Division of Urology, Tohoku Medical and Pharmaceutical University, Sendai, Japan

**Keywords:** case reports, internal hernia, intestinal obstruction, retroperitoneal neoplasm/surgery, retroperitoneal space, transmesenteric hernia

## Abstract

**Introduction and importance:**

Internal hernias following retroperitoneal surgery are rare but potentially life-threatening. We report a case of intestinal obstruction caused by an internal hernia with dual hernia orifices after laparoscopic retroperitoneal tumor resection, highlighting the unique pathogenesis and the importance of mesenteric defect closure in retroperitoneal surgery.

**Presentation of case:**

A 60-year-old female patient underwent laparoscopic transabdominal resection of a retroperitoneal tumor located in the lower pole of the left kidney. On postoperative day 23, the patient presented with vomiting and abdominal pain. Contrast-enhanced computed tomography revealed a closed-loop obstruction extending into the perirenal space, with congested and dilated small bowel within the abdominal cavity, suggesting an internal hernia through multiple orifices. Emergency laparotomy revealed small bowel incarceration in the perirenal space through the descending mesocolon and Gerota’s fascia. The herniated bowel adhered to the perirenal space, preventing spontaneous reduction. No intestinal necrosis was observed. Adhesiolysis and bowel reduction were performed, followed by suture closure of the mesenteric defect and omental patching of Gerota’s fascia defect. No hernia recurrence was observed 4 months postoperatively.

**Clinical discussion:**

Dual hernia orifices involving the mesentery and Gerota’s fascia led to bowel entrapment and intestinal obstruction. Adhesion of the leading bowel segment within the perirenal space may have hindered spontaneous reduction, thereby promoting bowel ischemia.

**Conclusion:**

In laparoscopic retroperitoneal tumor resection, combined mesenteric and retroperitoneal defects are an important risk factor for intestinal obstruction due to internal hernia.

## Introduction

Internal hernia, occurring when intra-abdominal organs protrude through peritoneal or mesenteric defects into abnormal intra-abdominal compartments, is relatively rare, accounting for 0.6–5.8% of intestinal obstructions^[^1,2^]^.

Retroperitoneal internal hernia (RIH) is an even rarer condition in which the small bowel herniates into the retroperitoneal space through defects in the mesentery and/or Gerota’s fascia^[^3^]^. Symptoms are nonspecific and resemble those of general intestinal obstruction, including abdominal pain, nausea, and vomiting. As the herniated bowel lies within the retroperitoneum, physical findings are often subtle, with limited rebound or focal tenderness.

HIGHLIGHTS

- Retroperitoneal surgery can create multiple internal hernia pathways.
- Dual defects of the mesocolon and Gerota’s fascia caused strangulation.
- Computed tomography showing perirenal closed-loop obstruction suggests complex RIH.
- Adhesion within the retroperitoneum may prevent spontaneous reduction.
- Careful inspection and closure of mesenteric defects are essential.

Retroperitoneal procedures, such as nephrectomy, may create both mesenteric defects and opened retroperitoneal cavities, generating multiple potential hernia pathways and predisposing patients to complex, potentially strangulated obstruction^[^4^]^.

Computed tomography (CT) has a reported sensitivity and specificity of 82.0% and 84.8%, respectively, for diagnosing internal hernias. However, RIH can show atypical features that are easily missed^[^5^]^. Delayed diagnosis risks intestinal necrosis and high mortality, underscoring the importance of early recognition and urgent intervention.

This case study adds to the literature by demonstrating a dual-orifice internal hernia after laparoscopic retroperitoneal tumor resection, with sequential herniation through defects in the descending mesocolon and Gerota’s fascia. Moreover, the case study emphasizes meticulous inspection and closure of potential defects during retroperitoneal surgery.

This report follows the Surgical CAse REport (SCARE) guidelines^[^6^]^.

## Presentation of case

A 60-year-old Japanese female patient underwent laparoscopic tumor resection via a transabdominal approach for the treatment of a retroperitoneal tumor adjacent to the lower pole of the left kidney (Fig. 1A). The descending mesocolon was mobilized off Gerota’s fascia, which was widely incised to expose the lesion (Fig. 1B). The tumor, which was continuous with Gerota’s fascia caudal to the middle-lower pole of the kidney, was resected *en bloc* (Fig. 1C). Operative time was 1 h 55 min, with minimal blood loss. The postoperative course was uneventful, and the patient was discharged on postoperative day (POD) 4. Histopathology demonstrated capillary proliferation around degenerated tissue, consistent with hemangioma, without malignancy.

**Figure 1.**
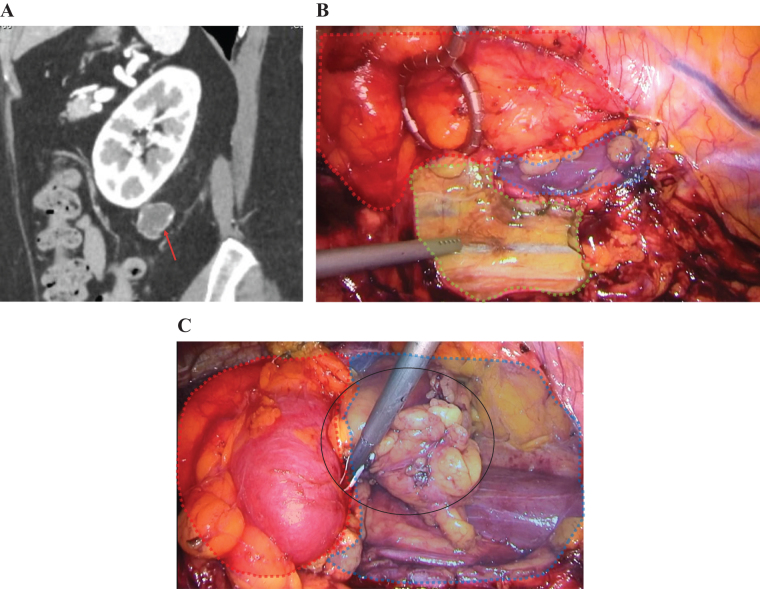
Preoperative and intraoperative imaging findings of the retroperitoneal tumor. (A) Sagittal contrast-enhanced computed tomography image showing a 23-mm nodule in the perirenal space at the caudal–dorsal aspect of the left kidney. (B) While retracting the kidney and retroperitoneal tumor ventrally using a snake retractor, Gerota’s fascia was incised, and the left kidney was mobilized. Red dotted line: left kidney and retroperitoneal tumor; green dotted line: descending mesocolon defect; blue dotted line: Gerota’s fascia defect exposing the iliopsoas muscle. (C) Separation of the kidney and retroperitoneal tumor. The red dotted line indicates the left kidney; the black circle represents the retroperitoneal tumor; the blue dotted line marks Gerota’s fascial defect exposing the iliopsoas muscle and ureter.

Her social history was notable for being unemployed, with full independence in daily activities. She had no history of smoking or alcohol use. Family history was unremarkable, and she had no known drug allergies.

On POD 23, the patient developed sudden periumbilical abdominal pain associated with nausea and more than 10 episodes of vomiting and visited the emergency department. Physical examination showed abdominal distension and periumbilical tenderness, without rebound tenderness or guarding.

Laboratory findings showed no significant abnormalities (white blood cell count: 13.6 × 10^3^/μL, hemoglobin: 16.5 g/dL, blood urea nitrogen: 22 mg/dL, creatinine: 0.71 mg/dL, and C-reactive protein: 0.11 mg/dL), except for a mildly elevated lactate (2.4 mmol/L).

Contrast-enhanced CT revealed a clear transition point with caliber change on the cranial and caudal sides at the caudal aspect of the lower pole of the left kidney, consistent with closed-loop obstruction rather than postoperative ileus (Fig. 2A–2C). No mesenteric whirl sign or twisting of mesenteric vessels suggestive of small bowel volvulus was noted. In addition, dilated small bowel, mesenteric edema, congestion, reduced wall enhancement, and ascites were observed within the perirenal space, separate from the closed loop (Fig. 2D). Although adhesive small bowel obstruction was considered, the retroperitoneal location of the transition point and the associated perirenal bowel/mesenteric congestion favored an internal hernia involving multiple hernial orifices and prompted the need for emergency laparotomy.

**Figure 2.**
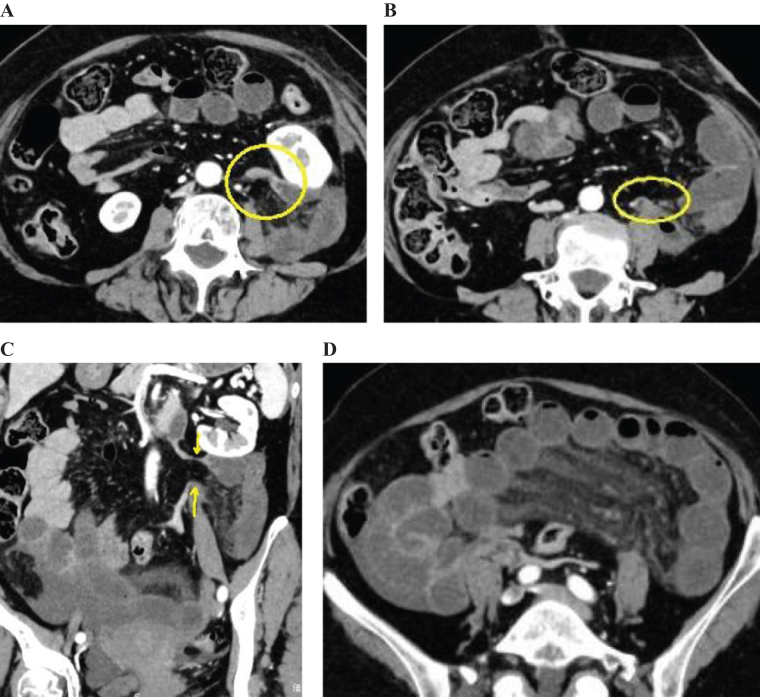
Contrast-enhanced computed tomography (CT) findings at the onset of an internal hernia. (A) The yellow circle indicates changes in the cranial caliber. (B) The yellow circle indicates a caudal caliber change. (C) Coronal view showing the two caliber changes at the caudal aspect of the lower pole of the left kidney, suggesting closed-loop formation in the perirenal space. Yellow arrows indicate cranial and caudal caliber changes. (D) Extensive congestion in the small intestine beyond the closed loop formed in the left perirenal space, suggesting another hernial orifice in addition to the perirenal space, although no other hernial orifice is evident on the CT.

Intraoperatively, an approximately 10-cm defect in the descending mesocolon and an approximately 5-cm defect in Gerota’s fascia were observed. Most of the bowel had herniated leftward through the mesenteric defect, with a segment incarcerated and adherent in the perirenal space (Fig. 3A). After sharp adhesiolysis, the bowel was reduced to the right side through the mesenteric defect (Fig. 3B). The mesenteric defect was closed using 4-0 polydioxanone suture (Johnson & Johnson MedTech, Cincinnati, OH, USA). The fascial defect was attributed to partial fascial resection during the prior retroperitoneal oncologic surgery; as the defect was too large to close without tension, an omental patch was applied (Fig. 3C). Although the bowel was congested, indocyanine green fluorescence imaging revealed preserved bowel perfusion, and bowel resection was deemed unnecessary (Fig. 3D). No anti-adhesion barriers were applied after irrigation. A closed-suction drain was placed in the left paracolic gutter, and the surgery was completed.

**Figure 3.**
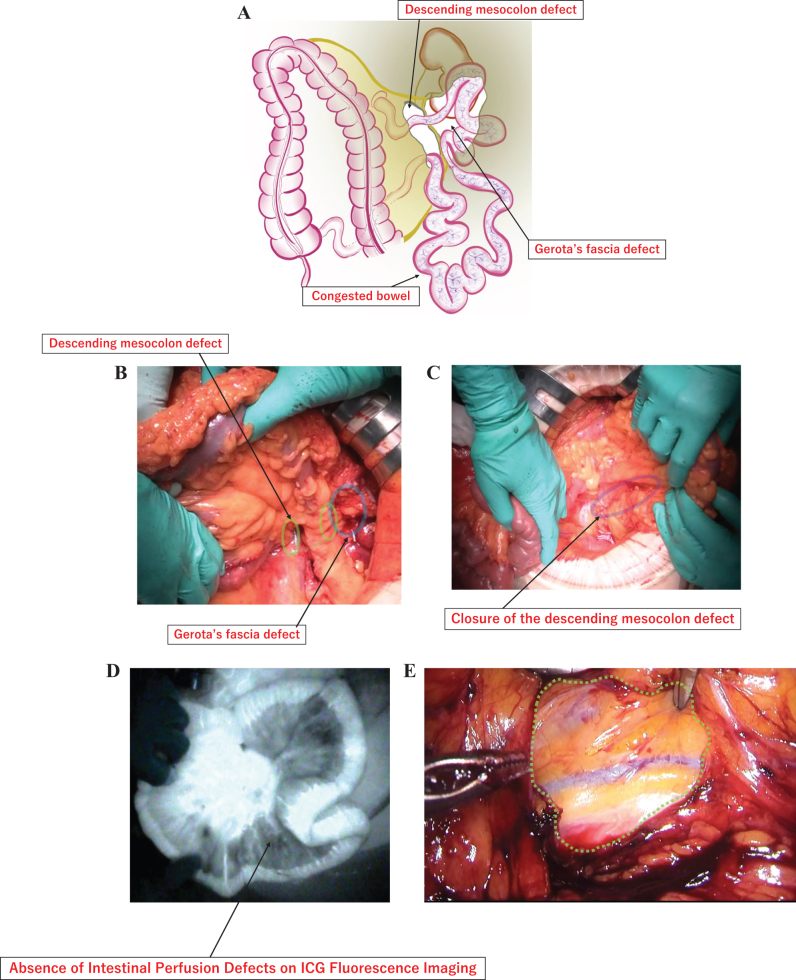
Intraoperative findings during open internal hernia repair surgery and descending mesocolon defect. (A) Schematic diagram of the open surgery showing the hernial orifices at the descending mesocolon and Gerota’s fascia defects, with congestion observed throughout the small intestine, which was incarcerated to the left of the descending mesocolon. (B) Intraoperative photograph after reduction of the entire small intestine, showing the hernia orifice dorsal to the descending colon with the exposed lower pole of the kidney through the Gerota’s fascia defect. (C) Surgical photograph showing closure of the descending mesocolon defect. (D) Indocyanine green (ICG) fluorescence imaging shows no obvious areas of reduced bowel wall enhancement. (E) Retrospectively, a defect in the descending mesocolon (green dotted line) was observed during dissection of the descending mesocolon from Gerota’s fascia.

On POD2, a contrast-enhanced CT was performed in response to persistently bloody drain output to evaluate for postoperative bleeding; it revealed a left retroperitoneal hematoma and a pulmonary embolism. The hematoma was managed conservatively, and the pulmonary embolism resolved with anticoagulation.

A follow-up was planned on an outpatient basis at 3-month intervals as part of surveillance after retroperitoneal tumor resection, with assessment of symptoms, bowel function, and social functioning, as well as blood tests and imaging studies when clinically indicated. At the 4-month follow-up after the initial laparoscopic surgery, no recurrence of the tumor or RIH was observed. Functional recovery was favorable: she was discharged home, and she remained independent in daily activities at the preoperative baseline. She reported no abdominal pain, and her dietary intake and bowel movements remained satisfactory.

## Discussion

### RIH pathogenesis

RIH occurs when the bowel passes through mesenteric defects into the retroperitoneal space. Three conditions are generally required: a mesenteric defect following transabdominal or retroperitoneal approaches; the formation of an open retroperitoneal space, often occurring in retroperitoneal surgeries such as nephrectomy; and sufficient bowel mobility due to limited adhesions^[^3^]^.

Although nephrectomy is a typical setting, a similar anatomy was created in our patient. Wide opening of Gerota’s fascia, together with a defect in the descending mesocolon, established dual pathways for RIH. Passage through the mesenteric defect, followed by fixation within the perirenal space, likely hindered spontaneous reduction and promoted strangulation.

### Internal hernia: diagnosis and treatment

CT is the first-line diagnostic modality. Typical findings include mesenteric swirl, abrupt mesenteric vein narrowing, mushroom-shaped hernial bulging, and reduced bowel wall enhancement^[^7^]^. In our case, a closed loop in the left perirenal space and reduced bowel wall enhancement supported the diagnosis. Ischemic bowel changes, separate from the loop, suggested multiple orifices.

Emergency surgery is often required. Although laparoscopy is minimally invasive, adequate visualization may be limited by bowel dilation or adhesions, and conversion rates of up to 32.4% have been reported^[^8^]^.

Key operative principles include identifying all defects, evaluating perfusion, and closing potential spaces. Our case had two orifices. As Gerota’s fascia defect was too large for primary closure, omental patching was performed.

Review of the original surgical video later demonstrated that the descending mesocolon defect had not been recognized as a hernia risk (Fig. 3E).

### Comparison with RIH after nephrectomy

Internal hernia is a rare but recognized complication after nephrectomy. After a review of 17 cases of internal hernia after laparoscopic nephrectomy, we observed that all cases occurred on the left side; five cases required bowel resection, and eight cases underwent laparotomy^[^3^]^. Herniation typically occurs through the descending mesocolon^[^9^]^.

In these reported cases, the hernia characteristically developed through a single mesocolic defect into the retroperitoneal space vacated by the resected kidney, with symptom onset ranging from 5 days to 8 weeks postoperatively. Unlike these reports, our case followed tumor resection rather than nephrectomy and involved two independent defects, illustrating that complex RIH can occur following other retroperitoneal procedures. The kidney was preserved, and the retroperitoneal space was created by partial Gerota’s fascia resection rather than organ removal, and the incarcerated bowel adhered to the perirenal space, preventing spontaneous reduction. Despite the dual-orifice configuration, bowel viability was preserved, and resection was not required, in contrast to five of the reported nephrectomy cases.

### Laparoscopy and prevention

The incidence of postoperative internal hernia has increased with the widespread adoption of laparoscopic surgery, reportedly 15- to 20-fold higher than that with open surgery^[^10,11^]^, and has also been described after laparoscopic distal pancreatectomy and pancreaticoduodenectomy^[^12,13^]^.

These findings suggest that laparoscopic surgery, with minimal adhesion and preserved bowel mobility, may increase the risk of postoperative internal hernia. In previous reports, RIH after nephrectomy has been reported predominantly following laparoscopic nephrectomy^[^3,9^]^, while reports following open nephrectomy are extremely rare^[^14^]^.

Moreover, among internal hernias after laparoscopic colonic resection, left-sided colectomy is the most common procedure, and defects in the descending mesocolon are most frequently identified^[^11^]^. Anatomically, the ligament of Treitz may predispose the small bowel to shift toward the left side, which could explain the higher incidence of internal hernia after left nephrectomy^[^15^]^.

In colonic cancer surgery, the mesenteric defects are not always closed, partly because laparoscopic closure can be technically challenging and, when performed under tension, may risk mesenteric tearing or compromise mesenteric perfusion. In contrast, retroperitoneal tumor resection or nephrectomy does not typically require oncologic mesenteric resection; therefore, any iatrogenic mesenteric defects should, in theory, be amenable to primary closure in many cases. In addition, disruption of Gerota’s fascia and dissection of an extensive perirenal space may act as dual hernia orifices, thereby creating a double risk for internal herniation. Therefore, mesenteric defects should be actively explored, carefully evaluated, and closed whenever possible, particularly during left-sided retroperitoneal surgery^[^3^]^.

Moreover, when complete or tension-free closure is difficult, small bowel adhesion within the perirenal space may promote bowel strangulation; therefore, adhesion barriers may be useful in selected cases. Finally, before completing retroperitoneal tumor resection or nephrectomy, the mesocolon should be carefully inspected for any defects, and meticulous handling during mobilization from Gerota’s fascia is essential to avoid iatrogenic mesenteric injury.

## Conclusions

To our knowledge, this is the first reported case of RIH occurring after laparoscopic retroperitoneal tumor resection, in which the small bowel was incarcerated in the perirenal space through dual hernia orifices. In cases of suspected internal hernia after laparoscopic retroperitoneal surgery, urgent CT should be performed to detect bowel ischemia, as complex internal hernias with multiple orifices may develop depending on the surgical approach and the extent of retroperitoneal dissection.

## Data Availability

The data that support the findings of this study are available from the corresponding author upon reasonable request.
